# HP promotes neutrophil inflammatory activation by regulating PFKFB2 in the glycolytic metabolism of sepsis

**DOI:** 10.1371/journal.pone.0296266

**Published:** 2024-01-16

**Authors:** Song Chen, Qian Zhang, Liyan Sun, Wei Song, Tao Zhang, Weidong Song, Jian Wan

**Affiliations:** Department of Emergency and Critical Care Medicine, Shanghai Pudong New Area People’s Hospital, Shanghai, China; The Hormel Institute (University of Minnesota), UNITED STATES

## Abstract

**Background:**

Sepsis, described as an inflammatory reaction to an infection, is a very social health problem with high mortality. This study aims to explore the new mechanism in the progression of sepsis.

**Methods:**

We downloaded the GSE69528 dataset to screen differentially expressed genes (DEGs) for WGCNA, in which the key module was identified and analyzed by DMNC algorithm, expression verification and ROC curve analysis to identify the hub gene. Furthermore, the hub gene was analyzed by immunoassay, and the potential mechanism of hub gene in neutrophils was investigated by *in vitro* experiments.

**Results:**

The turquoise module was the key module for sepsis in WGCNA on 94 DEGs. The top 20 genes of DMNC network were verified in GSE69528 and GSE9960, and 10 significant genes were obtained for ROC analysis. Based on the ROC curves, HP was considered the hub gene in sepsis, and its expression difference in sepsis and control groups was substantially significant. Further, it was demonstrated the knockdown of HP and PFKFB3 could suppress glycolysis and inflammatory cytokine levels in dHL-60 cell treated with LPS.

**Conclusion:**

In conclusion, HP is identified as a potential diagnostic indicator for sepsis patients, and HP promotes neutrophil inflammatory activation by regulating PFKFB2 in the glycolytic metabolism of sepsis confirmed by in vitro experiments. These will help us deepen the molecular mechanism of sepsis.

## Introduction

Sepsis is one of the most prevalent critical disorders, a condition of physiological, morphological, and biochemical abnormalities brought on by infection [1, 2], with an increasing incidence every year worldwide [3]. The diagnosis and treatment of sepsis are complicated by the non-specificity of sepsis symptoms and signs [4]. Therefore, clinical biomarkers are necessary for the early diagnosis of sepsis patients [5]. Presently, C-reactive protein, interleukin-6, and procalcitonin [6] are the three primary laboratory markers used to make a clinical diagnosis of sepsis [4, 7]. However, their sensitivity and specificity are still unsatisfactory [8].

In recent decades, as a result of the creation and use of gene chip technology, microarray technology and bioinformatics analysis have been employed to analyze disease-related differentially expressed genes (DEGs) widely, and explore key genes for disease diagnosis, treatment and prognosis [9–11]. The creation of gene networks and the identification of prospective essential molecular targets are made possible by a thorough examination of sepsis [12–14]. These molecular targets could offer a fresh perspective on the pathophysiology of sepsis and might be used to help identify sepsis patients earlier and treat them clinically.

This study firstly identified DEGs between uninfected healthy individuals and sepsis patients in GSE69528 dataset for the weighted gene co-expression network analysis (WGCNA) [15, 16], functional enrichment analysis, protein-protein interaction (PPI) network, expression verification and receiver operating characteristic (ROC) curves to obtain the hub gene. We also investigated the relation between hub gene and immune cells, and confirmed their potential mechanism by *in vitro* assays. Overall, the study offers new directions into the immune disturbances and enable the investigation of novel treatment targets in sepsis.

## Materials and methods

### Microarray data

This study used the mRNA microarray data from GSE69528 in the GEO database, including 28 groups of uninfected healthy samples and 83 groups of patients with sepsis samples. DEGs between sepsis and healthy controls was screened by GEO2R. To balance the identification of statistically significant genes, adjusted *P*-values and Benjamini and Hochberg false discovery rates were employed. “*P* < 0.01 and FC (fold change) > 1.5 or < 0.67” were defined as thresholds for DEGs. Heatmaps were generated using the pheatmap package in two dimensions.

### Weighted gene co-expression network analysis (WGCNA)

We built co-expression networks of DEGs by the “WGCNA” package in R. In this study, a power of β = 28 (scale-free R^2^ = 0.85) was chosen for the scale-free network. The connectedness of a gene network was then assessed by converting adjacency into a topological overlap matrix (TOM), which is the sum of a gene’s adjacency with all other genes produced by the network. Additionally, a TOM and an adjacency matrix were created from the gene expression matrix. It was determined what the associated TOM (1-TOM) dissimilarities were. The connection between an expression profile and each clinical trait was used to compute the gene significance (GS) for each expression profile. The relationship between each ME and the expression profile is known as module membership (MM). As a result, both measures revealed genes with considerable importance for clinical characteristics and MM.

### GO term, KEGG pathway and PPI network analysis

For the genes of the obtained key module, we utilized the "clusterProfiler" function package of the R language for the GO and KEGG analyses. The STRING online database (version 11; The Search Tool for the Retrieval of Interacting Genes; http://string-db.org)(16) was applied to find anticipated interactions between major module genes. The STRING database was employed to create the PPI network of DEGs. We identified the top 20 genes from key module using the DMNC algorithm of the cytoHubba plugin in Cytoscape.

### The expression verification and ROC curves analysis

Subsequently, we analyzed the levels of the top 20 genes in the two sepsis-related datasets (GSE69528 and GSE9960) using boxplots. The overlapping genes with significant differences in the two datasets were screened by the Venn diagram tool. Subsequently, ROC package was employed to draw ROC curves for the overlapping genes. The gene with a high Area Under Curve (AUC) value was the key gene (HP), indicating a good diagnostic ability.

### Immunoassay

The infiltrating abundance and fraction of 24 immune cells in the samples were acquired from Immune Cell Abundance Identifier (ImmuCellAI) (http://bioinfo.life.hust.edu.cn/web/ImmuCellAI/). In addition, the Spearman correlation of HP and immune cells was also analyzed.

### Cell culture and transfection

In RPMI-1640 medium containing 1% penicillin-streptomycin and 10% FBS, the neutrophils were resuspended before being stimulated for 12 hours with 10 μg/mL lipopolysaccharide (LPS) and tumor necrosis factor alpha (TNF-α). HL-60 cells were cultivated for 6 days in the presence of 1.25% dimethylsulfoxide (DMSO) in order to stimulate differentiation of neutrophil-like HL-60 (dHL-60) cells. After that, LPS was applied to the dHL-60 cells for 12 hours of stimulation. Subsequently, HP [small interfering (si)-HP-1 and si-HP-2] and overexpression (over-HP) were knocked down in LPS-induced dHL215 60 with Lipofectamine 2000 reagent, respectively, and transfected for a period of time before proceeding to the next step.

### Quantitative reverse-transcription PCR (qPCR) analysis

The measurement of gene expression analysis was conducted using qRT-PCR. The total RNA was extracted from neutrophils using the TRIzol reagent and RNeasy kit. A spectrophotometer was used to measure the purity of the RNA, and denaturing agarose gel electrophoresis was applied to evaluate the integrity of the RNA. Subsequently, RT-qPCR was conducted using the one-step SYBR Premix Ex Taq (Roche). The following primer sets were used, HP, forward, 5’-CTGTGCrGGCATCTCTAAG-3’ and reverse, 5’-CAGCTATGATCTTCTGAAC-3’; PFKFB2, forward, 5’-ATTGCGGTTTTCGATGCCAC-3’ and reverse, 5’-GCCACAACTGTAGGGTCGT-3’.

### Western blotting (WB) analysis

To detect the protein of interest, cells were treated with RIPA buffer supplemented with a protease inhibitor cocktail. Following SDS electrophoresis on a 10% polyacrylamide gel, the resultant cell lysates were deposited onto a nitrocellulose membrane. After that, a rabbit anti-PFKFB2 monoclonal antibody was incubated on the membrane for an entire night at 4°C, followed by an hour-long incubation with the secondary antibody indoor. Finally, the bands were visualized by enhanced chemiluminescence (ECL) reagents and Hyper Thin Film ECL. The intensity was measured using ImageJ software.

### The enzyme-linked immunosorbent assay (ELISA)

To detect the presence of inflammatory factors, an ELISA multiplex assay was performed. A 96-well plate was washed with wash buffer for 10 minutes on a shaker, then dried, and 50 μl of conjugated beads were added to each well. Standards and samples were added to the appropriate wells and kept for 1 hour with agitation on a plate shaker. The plate was subsequently washed twice, and 25 μl of detection antibody was added to the wells, followed by incubation with agitation for 30 minutes, wrapped in foil, at 20–25°C. Then, a streptavidin-phycoerythrin solution of 50μl /well was added. The beads were resuspended in assay buffer and the plate rinsed twice after 10 minutes. Finally, the Bio-Plex 200 system was used to assess the quantity of inflammatory components.

### Statistical analysis

All experiments in this study were conducted independently and in triplicate. All analyses were performed using spss 19.0 software. Data were shown as mean ± standard deviation (SD). Student’s unpaired t-test was used to determine significant differences in other outcomes. These data were considered statistically significant; "*" indicates "P < 0.05", "**" indicates "P < 0.01 ", and "***" indicates "P < 0.001".

## Results

### The turquoise module was the key module in WGCNA on GSE69528-DEGs

**Fig 1:**
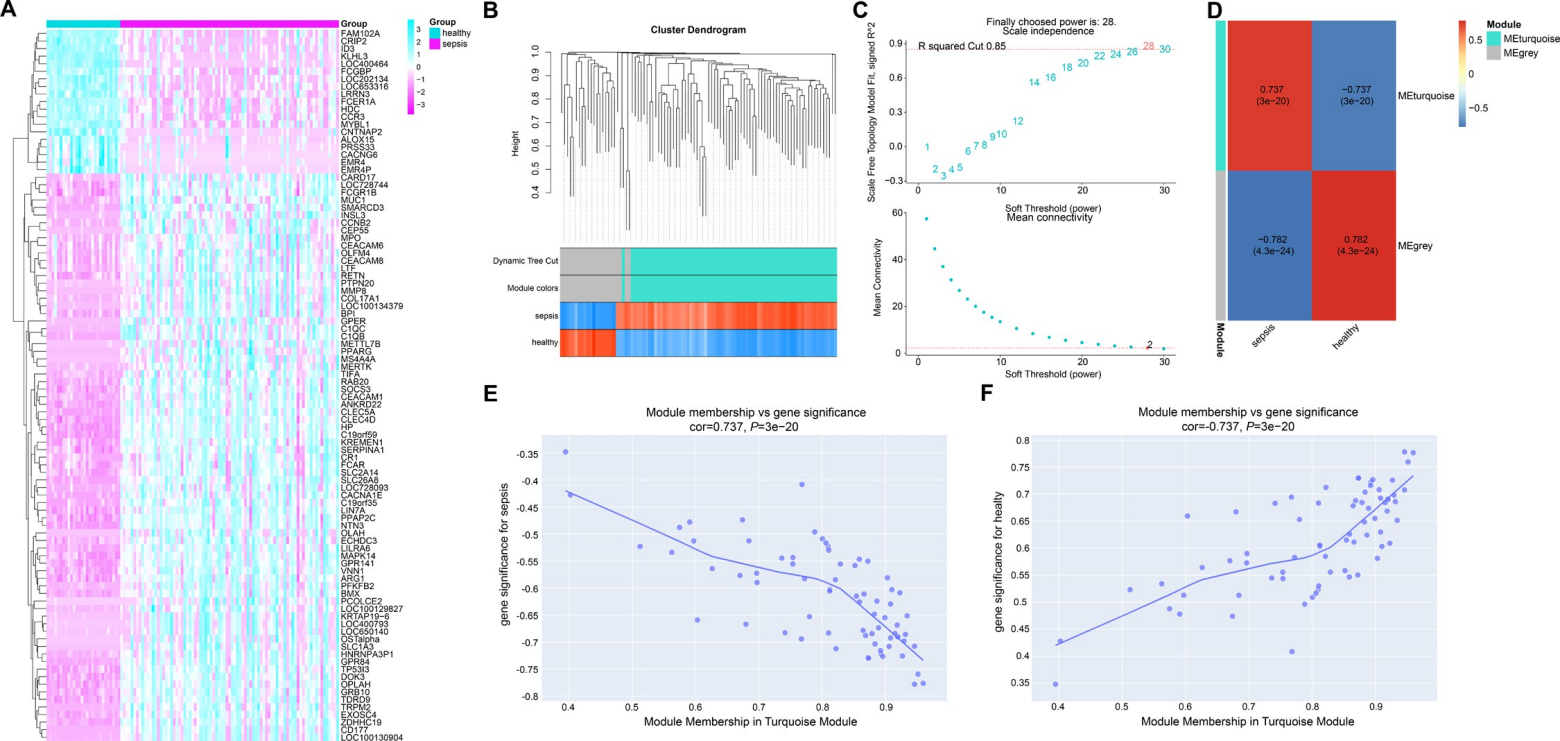
The turquoise module was the key module in WGCNA on GSE69528-DEGs. (A) Heatmap analysis of identified DEGs between septic patients and uninfected controls. Red shows down-regulated DEGs, and blue shows up-regulated DEGs. (B) Dendrogram of different gene clusters based on the topological overlap, and assigned module colors. (C) Effect of different soft threshold power values on scale independence and average connectivity. (D) Module feature relationship construction. Each row corresponds to an ME, and each column corresponds to a feature. (E, F) Scatterplots of module membership versus gene importance in the turquoise module.

According to the screening criteria, 75 up-regulated and 19 down-regulated DEGs were identified. The heatmaps of the identified DEGs revealed significant differences between the sepsis and control groups (Fig 1A). The average linkage hierarchical clustering approach was applied to group the included samples (Fig 1B). The soft threshold parameter for the scale-free network was set to a power of 28, and 2 modules were identified by mean-linkage hierarchical clustering. The heatmap of module-trait interaction was created to evaluate the link between each module and two clinical characteristics (sepsis and health, Fig 1D). The turquoise module had a close relation with sepsis (r = 0.737, *P* = 3e-20). Scatterplots of GS versus MM in turquoise module were demonstrated in Fig 1E and 1F. According to the aforementioned findings, there was a strong association between the turquoise module and sepsis clinical features.

### The enrichment analysis of genes in turquoise module

**Fig 2:**
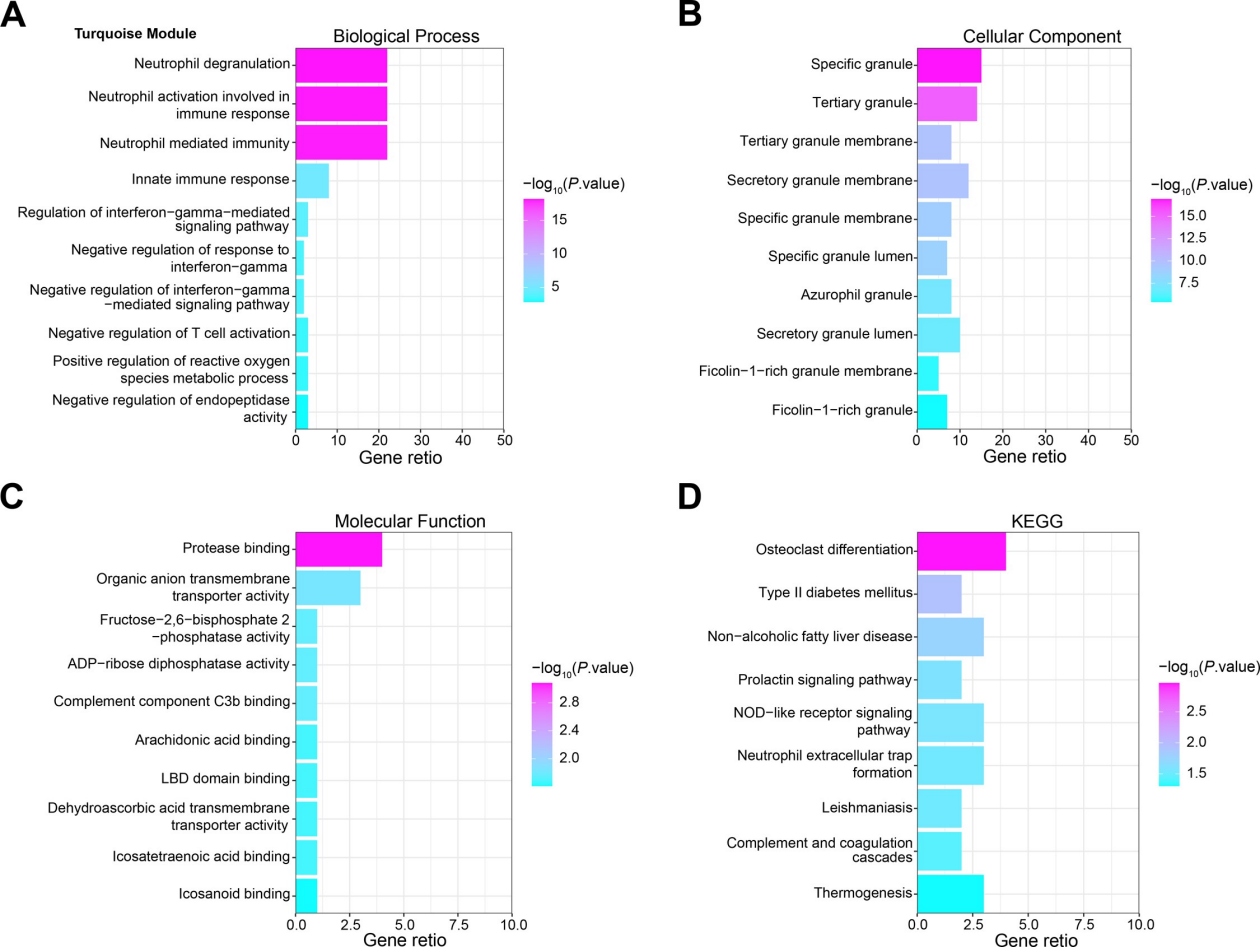
GO and KEGG enrichment analysis of genes in the turquoise module. (A) The top 10 important biological process terms of the turquoise module genes were enriched. (B) The top 10 significant cellular component terms of turquoise module genes are enriched. (C) The top 10 important molecular function terms of the turquoise module genes were enriched. (D) Enrichment results of the 10 KEGG pathways with the largest number of genes. The horizontal axis in the figure represents the number of enriched genes, and the vertical axis represents the name of each KEGG pathway.

Through the ClusterProfiler package in R analysis, the GO terms enriched by genes in turquoise module mainly included Neutrophil degranulation, Specific granule, Neutrophil activation involved in immune response, Tertiary granule, Protease binding and Organic anion transmembrane transporter activity, etc. (Fig 2A–2C). The KEGG pathways of turquoise module genes mainly contained Osteoclast differentiation and Type II diabetes mellitus, etc. (Fig 2D).

### The hub gene related to sepsis was HP in this study

**Fig 3:**
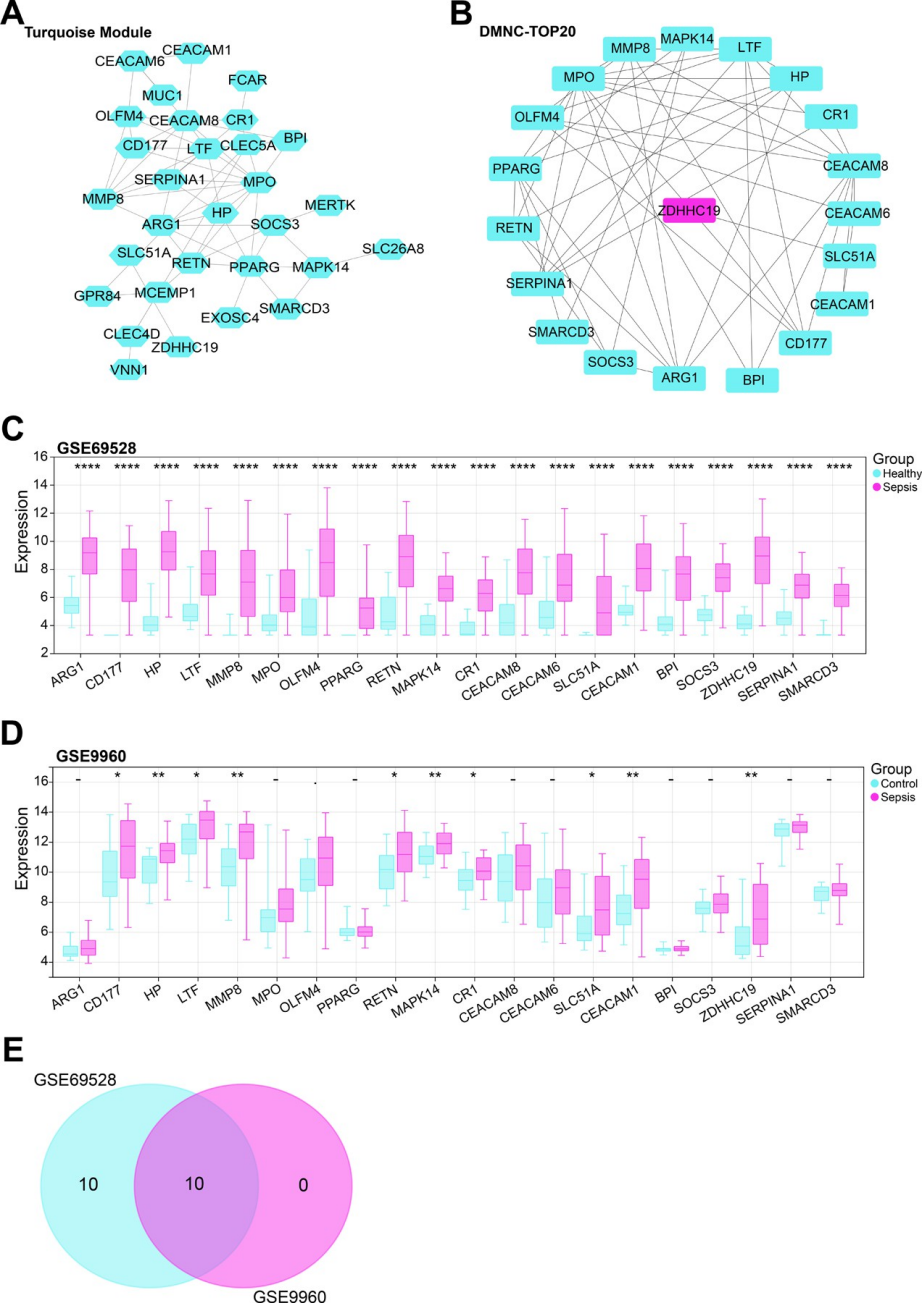
PPI network analysis and expression analysis of turquoise module genes. (A) PPI network map of sepsis-related key module genes. Each dot represents a node and lines represent connectivity between genes. (B) Top 20 hub gene centers identified by the density of maximum neighborhood component (DMNC) density. (C) Expression levels of 20 genes in sepsis samples and healthy samples in the GSE69528 dataset. (D) Expression levels of 20 genes in sepsis samples and healthy samples in the GSE9960 dataset. (E) Venn diagram showing overlapping genes with differentially significant genes. *P<0.05, **P<0.01, ****P<0.0001.

**Fig 4:**
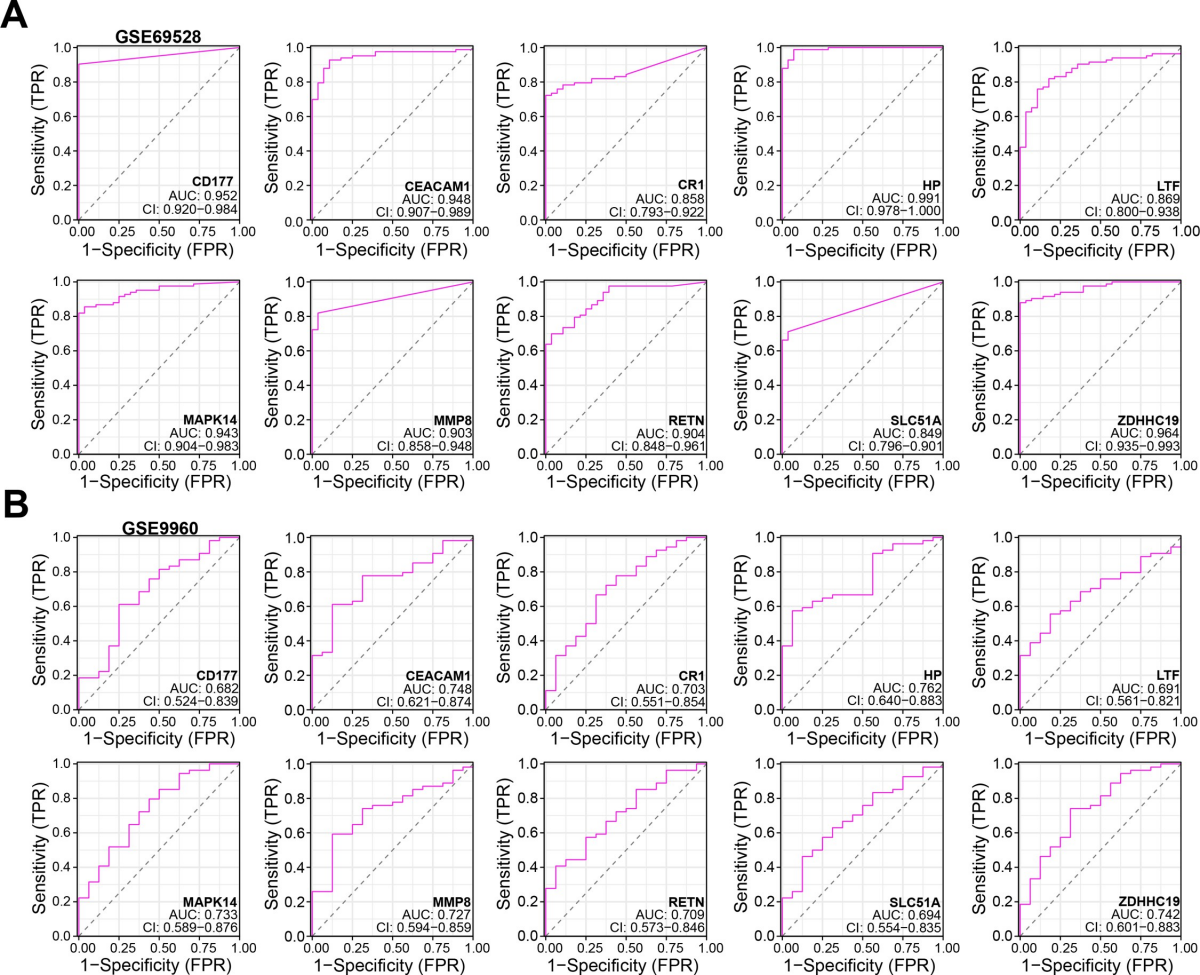
Receiver operating characteristic (ROC) curve analysis on overlapping genes. (A) ROC curves of 10 crossover genes in the GSE69528 dataset. (B) ROC curves of 10 crossover genes in the GSE9960 dataset.

The STRING database was employed to build the PPI network of genes in the turquoise module (Fig 3A). By the DMNC algorithm of the CytoHubba plugin, top 20 genes were selected from the network (Fig 3B). In the GSE69528 dataset, the expression results of 20 genes were all significant (Fig 3C). In the GSE9960 dataset, the expression results of 10 genes were significant (Fig 3D). Thus, 10 overlapping genes with significant expression were identified by Venn diagram (Fig 3E). According to the ROC curve analysis of the 10 genes in the two datasets, we found that the AUC value of the HP was the highest, indicating a good diagnostic value in sepsis (Fig 4). Therefore, HP was targeted for the next analysis.

### The immunoassay results of HP in sepsis

**Fig 5:**
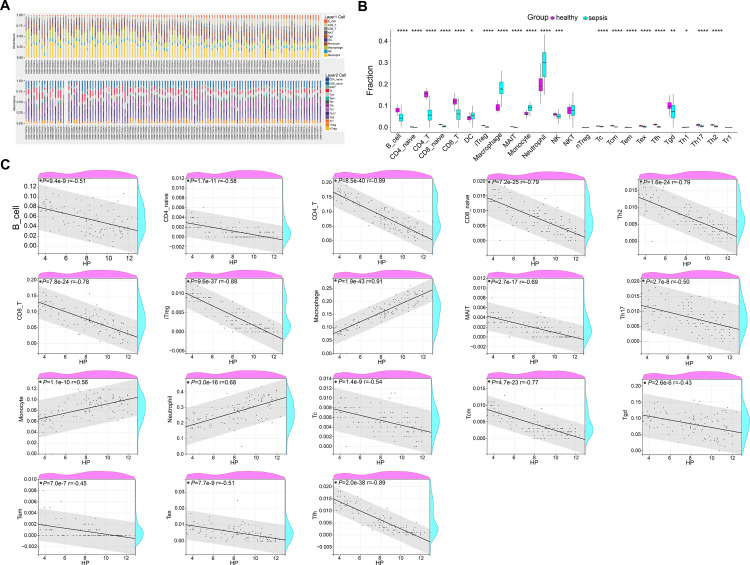
The immunoassay on HP in sepsis. (A) The expression abundance of 24 immune cells in 111 samples. (B) Expression scores of 24 immune cells in sepsis samples and healthy samples. (C) Spearman correlation analysis of immune cells and HP expression levels. *P<0.05, **P<0.01, ****P<0.0001.

In Fig 5A, the expression abundance of 24 immune genes was shown in 111 samples, and the expression scores of 24 immune genes in sepsis and healthy samples were demonstrated in Fig 5B. From the correlation analysis, B cell, CD4 naive, CD8 T, CD4 T, CD8 naive, Treg, MAIT, Tc, Tcm, Tem, Tex, Tfh, Tgd, Th2 and Th17 had a negative relation with the expression of HP. Macrophage, Monocyte and Neutrophil had a positive relation with the expression of HP (Fig 5C). HP had a strong relation with immune cells Macrophage and CD4 T. Notably, the expression of HP was significantly higher in neutrophils. In addition, studies have shown that human HP promotes breast cancer by regulating glycolysis activity. The primary metabolic pathway for pathogen clearance by neutrophils is glycolysis. In the GSE69528-DEGs, PFKFB2 is revealed to regulate glycolysis and cell growth of pancreatic cancer. Thus, we suspect that there is certain relation among HP, PFKFB2, glycolysis and neutrophils in sepsis.

### HP and PFKFB2 were up-regulated in dHL-60 treated with LPS

**Fig 6:**
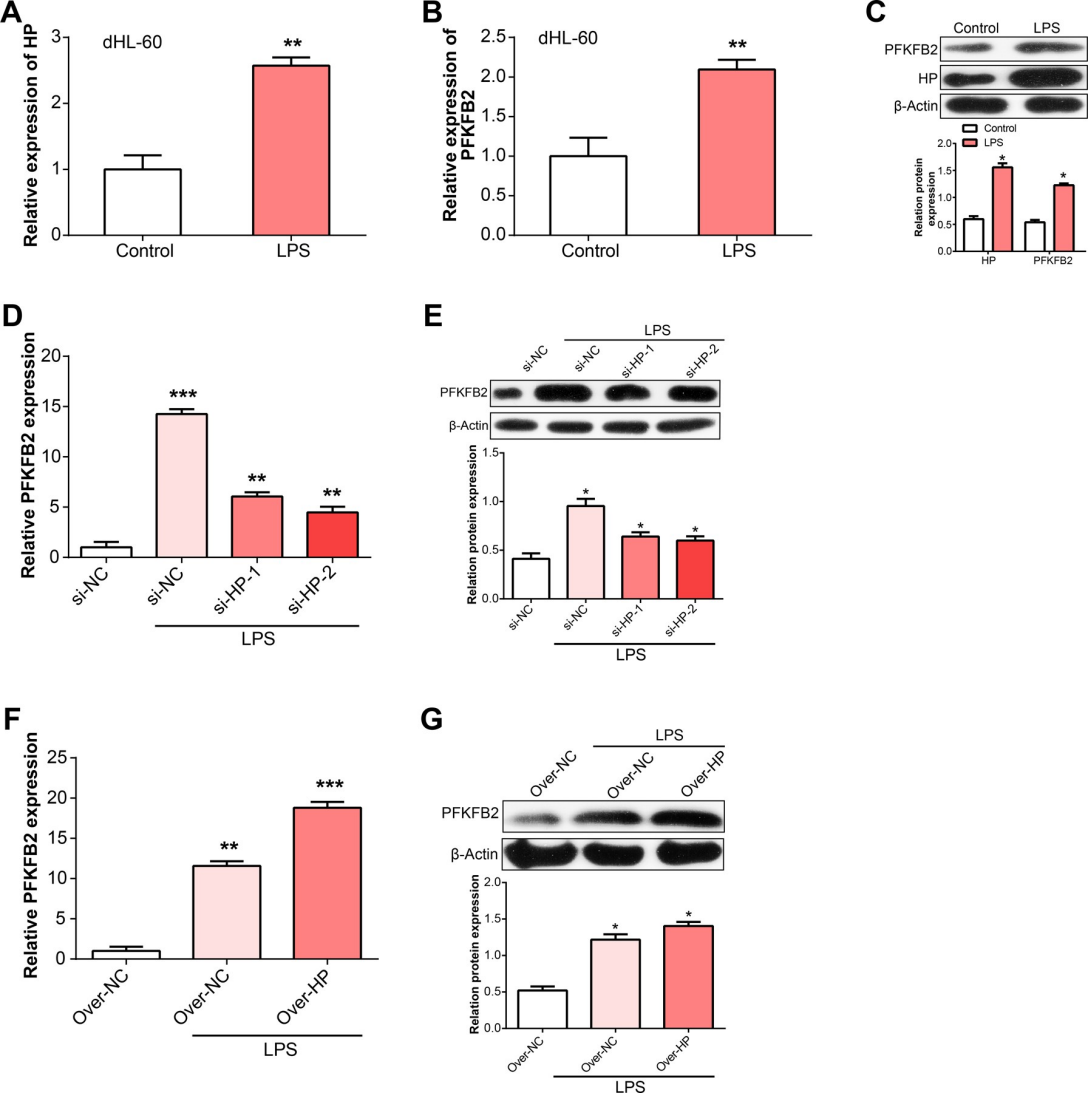
HP and PFKFB2 were up-regulated in dHL-60 treated with LPS. (A) Expression levels of HP in dHL-60 cells treated with LPS. (B) Expression levels of PFKFB3 in dHL-60 cells treated with LPS. (C) Western blot analysis of HP and PFKFB3 in dHL-60 cells treated with LPS. The bar graph on the side shows the results of the gray-scale detection of proteins. (D) HP knockdown reduced the expression level of PFKFB3 in dHL-60 cells treated with LPS. (E) Western blot analysis of PFKFB3 in HP knockdown dHL-60 cells treated with LPS. The bar graph on the side shows the results of the gray-scale detection of proteins. (F) Over-expression of HP increased PFKFB3 mRNA level in dHL-60 cells treated with LPS. (G) Western blot analysis of PFKFB3 in dHL-60 cells treated with LPS and over-HP. Neutrophils and dHL-60 cells were treated with LPS for 12 hours. The bar graph on the side shows the results of the gray-scale detection of proteins. **P <0.01, ***P <0.001.

Next, we detected the levels of HP and PFKFB2 in dHL-60 treated with LPS, which resembled neutrophils. The results showed the mRNA and protein levels of HP and PFKFB2 were both up-regulated in sepsis (Fig 6A–6C). Next, we generated dHL-60 cells with stable HP knockdown (si-HP-1, si-HP-2)/overexpression (over-HP) using lentiviral vector and an empty vector as a control. Our findings suggested the mRNA and protein expressions of PFKFB2 was significantly decreased by HP knockdown (Fig 6D and 6E), while increased by HP over-expression (Fig 6F and 6G). These results indicate HP plays a vital role in controlling the level of PFKFB2 in response to LPS stimulation in neutrophils.

### Glycolytic metabolism supported by HP/PFKFB2 can enhance the expression of inflammatory cytokines

**Fig 7:**
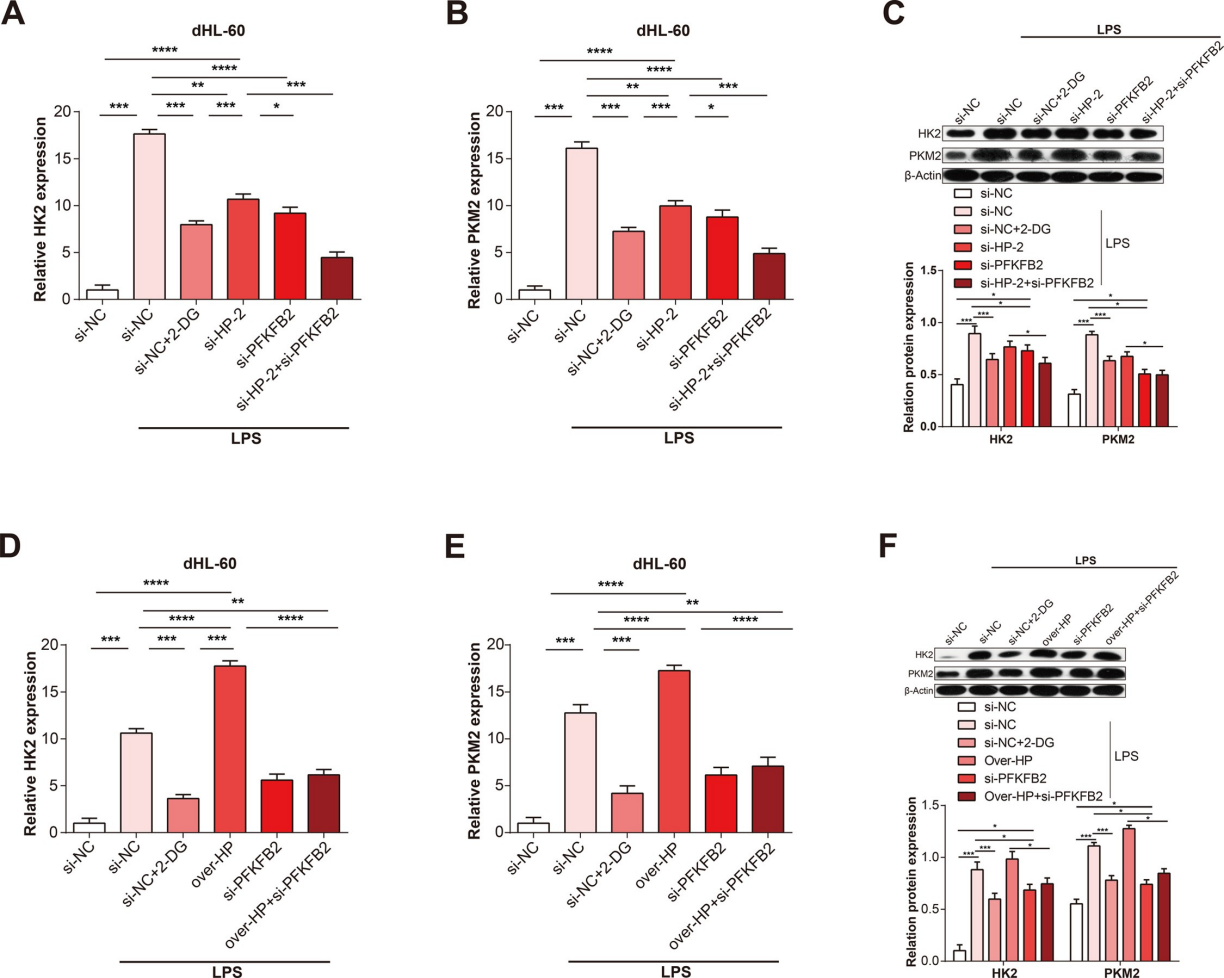
Glycolytic metabolism was supported by HP and PFKFB2 in sepsis. (A-C) qRT-PCR and WB detection of HK2 and PKM2 expression in dHL-60 cells with si-HP-2+si-PFKFB2, compared to control group. The bar graph on the side shows the results of the gray-scale detection of proteins. (D-F) qRT-PCR and WB detection of HK2 and PKM2 expression in dHL-60 cells with over-HP+si-PFKFB2, compared to control group. The bar graph on the side shows the results of the gray-scale detection of proteins. *P<0.05, **P<0.01, ***P<0.001, ****P<0.0001.

**Fig 8:**
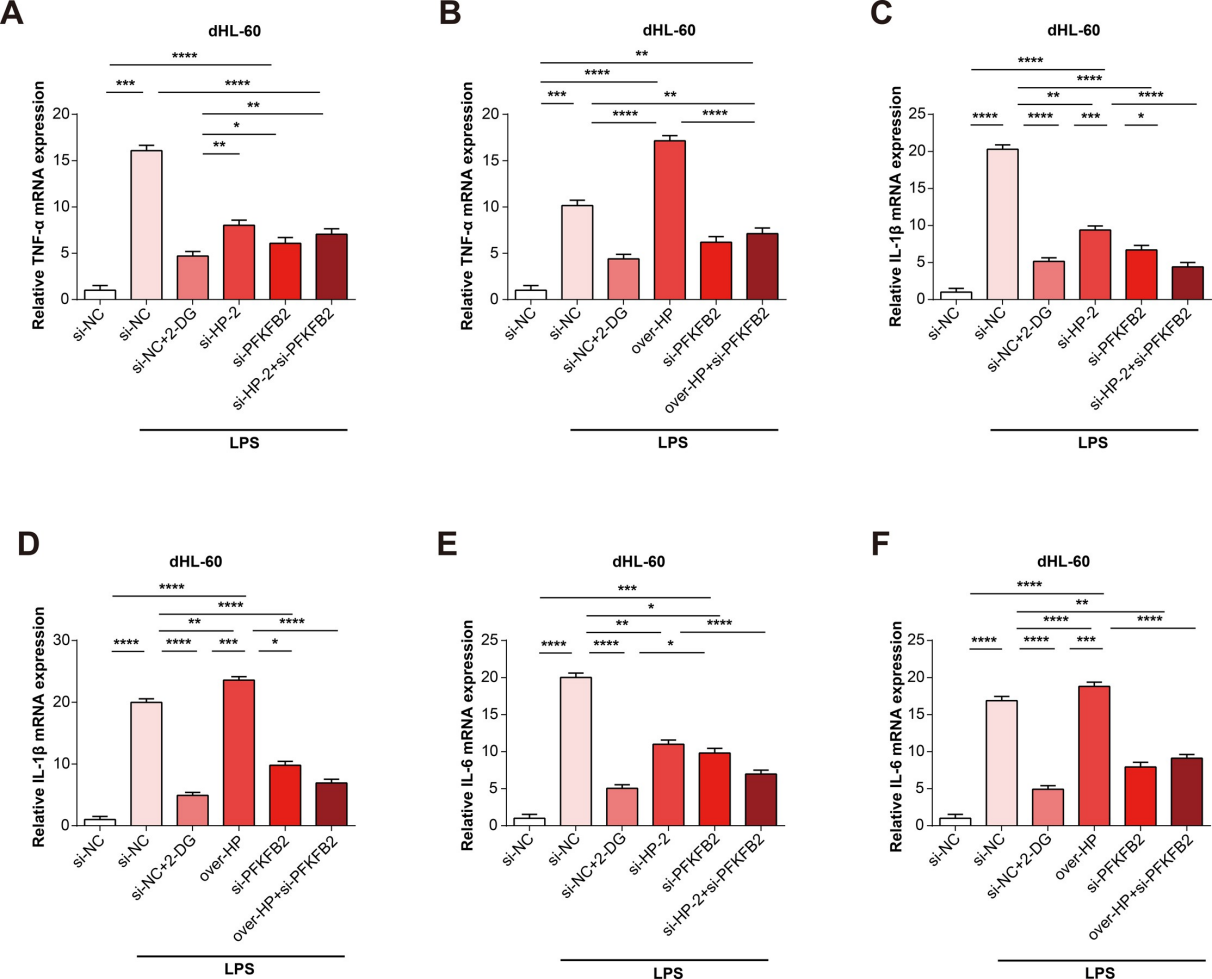
HP and PFKFB2 could promote the mRNA expressions of inflammatory cytokines. (A-B) The effect of si-HP-2+si-PFKFB2 and over-HP+si-PFKFB2 on TNF-α in dHL-60 cells with LPS detected by qRT-PCR. (C-D) The effect of si-HP-2+si-PFKFB2 and over-HP+si-PFKFB2 on IL-1β in dHL-60 cells with LPS detected by qRT-PCR. (E-F) The effect of si-HP-2+si-PFKFB2 and over-HP+si-PFKFB2 on IL-6 in dHL-60 cells with LPS detected by qRT-PCR. *P<0.05, **P<0.01, ***P<0.001, ****P<0.0001.

**Fig 9:**
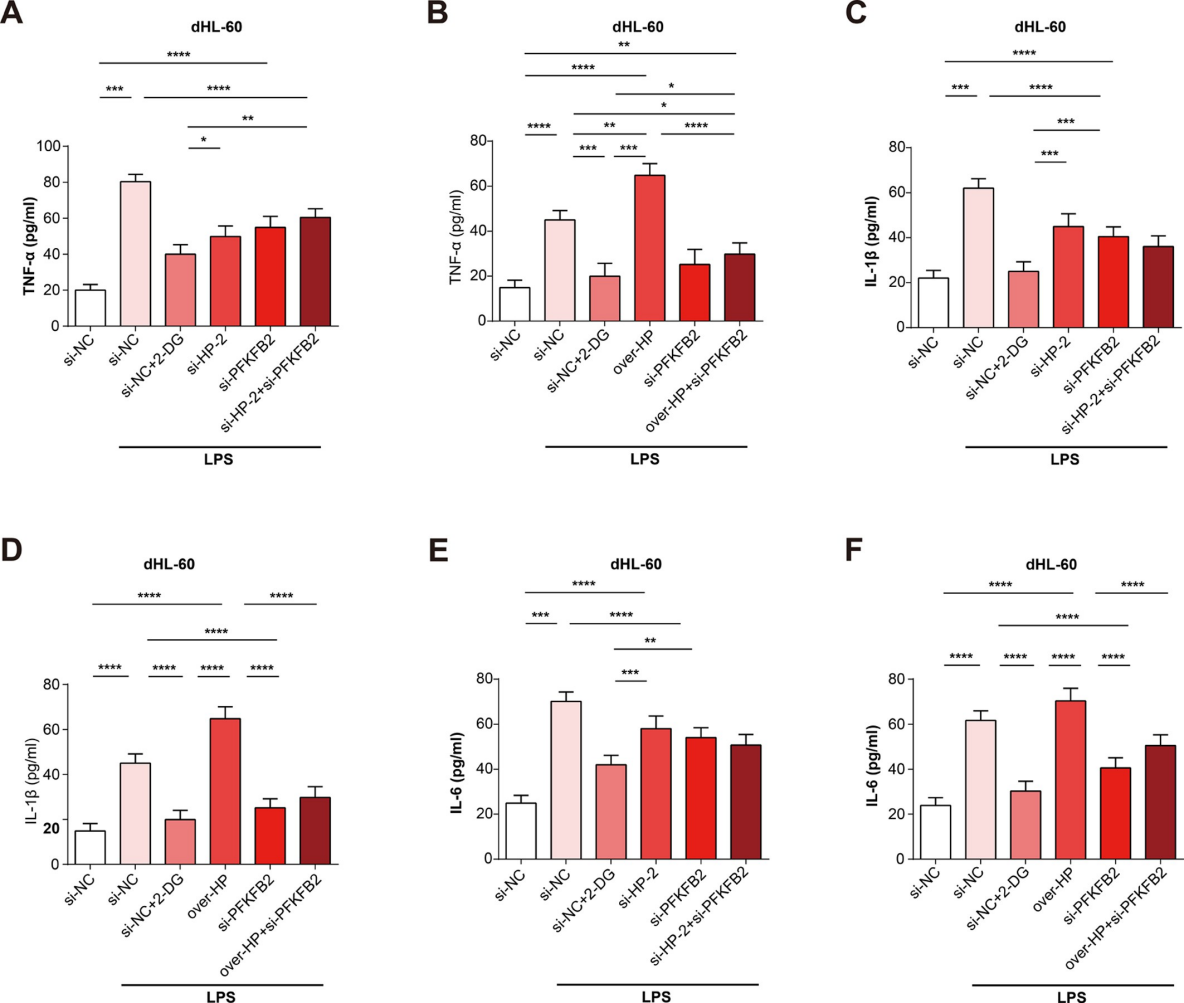
HP and PFKFB2 could promote the protein expressions of inflammatory cytokines. (A-B) The effect of si-HP-2+si-PFKFB2 and over-HP+si-PFKFB2 on TNF-α in dHL-60 cells with LPS detected by ELISA. (C-D) The effect of si-HP-2+si-PFKFB2 and over-HP+si-PFKFB2 on IL-1β in dHL-60 cells with LPS detected by ELISA. (E-F) The effect of si-HP-2+si-PFKFB2 and over-HP+si-PFKFB2 on IL-6 in dHL-60 cells with LPS detected by ELISA. *P<0.05, **P<0.01, ***P<0.001, ****P<0.0001.

To investigate whether HP regulates the glycolytic metabolism by PFKFB2, we measured the effect of si-HP-2+si-PFKFB2 and over-HP+si-PFKFB2 on the expressions of glycolysis indicators (HK2 and PKM2) in dHL-60 cells treated with LPS. it was found a significant reduction in the mRNA and protein expressions of HK2 and PKM2 by si-HP+si-PFKFB2 (Fig 7A–7C), while they were slightly increased by over-HP+si-PFKFB2 compared with si-PFKFB2 group (Fig 7D–7F). Furthermore, we investigated whether HP regulates the expression of inflammatory cytokines (TNF-α, IL-1β, and IL-6) through its involvement in PFKFB2-mediated glycolysis. To evaluate it, we assessed the mRNA expression level changes of inflammatory cytokines by si-HP-2+si-PFKFB2 and over-HP+si-PFKFB2 using qRT-PCR. We observed a significant reduction in inflammatory cytokine expressions in cells with knocked-down HP (Fig 8A–8F). Furthermore, the protein levels of inflammatory cytokines, determined by ELISA, were similar to the above results (Fig 9A-9F). These findings offered new directions the potential role of HP and PFKFB2 in regulating inflammatory responses, which could be explored further in the development of new therapeutic interventions.

## Discussion

In critical care medicine, sepsis is among the most frequent causes of mortality [17]. Sepsis is a very complicated pathophysiology that involves host immune reaction, pathogen invasion, and diverse tissue damage brought on by their intricate interactions [18, 19]. Despite extensive research on sepsis, the mortality rate from sepsis remains high. This could be the lack of reliable biomarkers for sepsis that can be used to diagnose and treat the condition early [20]. The molecular basis of disease incidence and progression may be understood through bioinformatics analysis, which also offers a fresh, efficient method for locating possible biomarkers for early diagnostic and treatment targets.

In this investigation, we explore genes that could be clinical biomarkers for sepsis patients. To obtain the important sepsis genes, we studied the GSE69528 and obtained a total of 94 DEGs between healthy samples and sepsis samples. WGCNA analysis of DEGs revealed that the turquoise module was a key module correlated with sepsis, mainly enriched in Innate immune response, Neutrophil mediated immunity and so on. Cells in the innate and adaptive immune systems play key roles in the host’s response to sepsis. Rimmelé, T et al. show that cells of the innate and adaptive immune systems play a key role in sepsis syndrome [21]. In addition, studies by Qiao Y et al. have shown that as a result of their intricate connections with vascular cells, neutrophils are essential to innate immunity, and systemic tissue injury may be associated with their activation [22]. The activation also results in the release of neutrophil traps that are essential for phagocytosis, pathogen containment, and coagulation activation. Numerous studies have demonstrated that sepsis patients usually have neutrophils that are expressed at quite high levels [23–25].

Next, we employed Cytoscape software and the DMNC algorithm to obtain the top 20 genes in the PPI network. Then, we analyzed their expression levels in the GSE69528 and GSE9960 datasets to find significant genes, respectively. Through the Venn diagram, 10 overlapping significant genes were screened out. ROC curve analysis of these genes showed that HP had the best diagnostic value, thus we targeted HP in the following analysis. Haptoglobin (HP) is a glycoprotein found in plasma with a crucial role in tissue protection and protection from oxidative damage. Its measurement is a part of routine clinical practice, as it is an acute-phase protein that undergoes changes in plasma concentration during pathological conditions [26, 27]. HP, a conserved protein synthesized primarily in the liver and lung, is being investigated as a potential biomarker for various diseases, including different forms of malignancy [28]. Research conducted by Isaac K Quaye et al. has demonstrated that HP can clear hemoglobin during intravascular or extravascular hemolysis, a protein function that is closely associated with inflammatory diseases [29]. Moreover, Graves, K. L et al. have reported in their study that HP not only serves as an essential antioxidant in vascular inflammation and atherosclerosis but also enhances inflammation in cardiac transplantation [30]. Our study revealed that HP was upregulated in sepsis samples and demonstrated good diagnostic value, making it an ideal biomarker for sepsis.

In our immunoassay, HP had a strong relation with immune cells Macrophage and CD4 T. Studies by Liu, Y. C et al. pointed out that the transformation of different phenotypes of macrophages regulates the initiation, development and arrest of inflammatory diseases [31]. As an escape cell that changes its phenotype in various microenvironments, macrophages may participate in the development of an immunosuppressive state in the sepsis response through polarization to the M2 phenotype [32]. Furthermore, data from Schmoeckel, K et al. showed in sepsis, a T cell receptor-independent route induces partial activation of CD4 T cells, but completes stimulation and proliferation need certain antigens [33]. Besides, the HP expression of sepsis and control groups was significantly high in neutrophils. The majority of innate immune cells, neutrophils, are the first mediators to reach the infection-affected region. Numerous investigations have demonstrated that the main metabolic mechanism for neutrophil-mediated pathogen clearance is glycolysis [34]. Glycolytic metabolism is initiated with the absorption of extracellular glucose, which is subsequently processed in the cytosol, ultimately yielding adenosine triphosphate (ATP) and other metabolites [35]. The above findings might be involved in the regulation of HP in sepsis.

Recent studies have reported that PFKFB2-mediated glycolysis is related to the regulation of inflammatory responses, and this gene was also found in the GSE69528-DEGs. PFKFB2 is a key enzyme in glycolysis that regulates the level of fructose-2,6-bisphosphate [36]. Dysregulation of PFKFB2 has been linked to several metabolic disorders, including diabetes [37], obesity [37], and cancer [38], making it a promising therapeutic target for these diseases. We suspected that PFKFB2 might be the target gene of HP in the glycolysis of sepsis. In the cell experiments, it was found increased expression of HP and PFKFB3 in sepsis, and there existed a positive relation between them. Besides, HP and PFKFB3 knockdown could regulated the levels of HK2 and PKM2, key indicators of glycolysis. Furthermore, we observed HP, an acute phase protein that is overexpressed during inflammation, promoted the level of inflammatory cytokines through PFKFB3-mediated glycolytic reprogramming. Given the pro-inflammatory effect of HP, our research reveals that HP-regulated PFKFB3-mediated glycolytic reprogramming is a possible sepsis treatment target.

## Conclusions

In summary, HP is up-regulated in sepsis with a strong diagnostic value, and it can serve as a reliable biomarker for sepsis diagnosis. Additionally, HP is involved in the immune response to sepsis. Through *in vitro* experiments, we demonstrate that HP enhances PFKFB3 transcription and translation, thereby promoting the production of neutrophil inflammatory cytokines in sepsis and playing a crucial role in promoting neutrophil glycolysis. Our study highlights the potential of key genes, such as HP and PFKFB3, to uncover novel regulatory pathways and identify key molecular targets for improving sepsis diagnosis, treatment and prognosis.
